# Antimicrobial prescription behavior in 16 German intensive care units: room for improvement in pneumonia therapy

**DOI:** 10.1186/2047-2994-4-S1-O2

**Published:** 2015-06-16

**Authors:** S Schneider, J Zweigner, F Schwab, M Behnke, E Meyer, P Gastmeier

**Affiliations:** 1 Hygiene Institute, Charité, Berlin, Germany; 2 Microbiology, Immunology, Hygiene, University Hospital, Cologne, Germany; 3 Department of Hygiene, Städtisches Klinikum, Munich, Germany

## Introduction

Antimicrobial surveillance in German hospitals is mainly based on consumption monitoring. Sparse data exist on prescription culture.

## Objectives

To investigate the performance of antimicrobial prescription behavior in order to identify starting points for interventions.

## Methods

16 intensive care units (ICUs) non-university hospitals covering Germany were included. Randomly selected medical records from patients diagnosed with pneumonia in 2012 were retrospectively analyzed. Bacteriological sampling and antimicrobial therapy (AMT) were reviewed with regard to clinically relevant aspects. Immunosuppressed, pregnant and underage patients were excluded.

## Results

383 medical records were analyzed (179 community acquired and 204 hospital acquired pneumonia cases). 40.5% of empiric therapy regimens were appropriate according to national guidelines. Regimens with too broad spectrum or not recommended substances were used in 11.7%. 47.8% had formally too narrow spectra. Duration of therapy could only be evaluated in 40.7% of cases since the other patients were dimissed or died before recommended end of therapy. Within the evaluable group 31.4% of therapies were too long. Dosing was adequate in 86.6% of cases. Bacteriological sampling was performed as recommended in 23.1% of cases and incompletely in 51.2%. Performance frequencies were 49.4% for blood cultures, 41.8% for tracheal aspirate and 15.4% for bronchoalveolar lavage. Legionella antigen was tested in only 7.6%. De-escalation strategies could be evaluated in 76.0% of cases, since 24.0% were dismissed before day 4. In 90% of evaluable cases no de-escalation was performed. In 6.9% de-escalation was microbiologically guided, in 3.1% clinically guided. Documentation of the indication for AMT was not performed in 25.5%. In 61.9% the indication was placed unsystematically in the medical record. Documentation at a predefined place was only found in 12.6%.

## Conclusion

Large room for improvement in AMT was detected regarding duration of therapy, performance of bacteriological sampling, de-escalation concepts and documentation. The high percentage of formally too narrow empiric AMT regimens should be interpreted carefully. Further analysis is needed to determine the best performance indicators.

## Disclosure of interest

None declared.

